# NECSO-based classification predicts immunotherapy efficacy and identifies FLAD1 as therapeutic target in kidney renal clear cell carcinoma

**DOI:** 10.3389/fimmu.2026.1914332

**Published:** 2026-07-28

**Authors:** Yitong Pan, Rui Wu, Xueyi Zhu, Xiaodi Hu, Jun Cheng, Lingwen Kong

**Affiliations:** 1 Hunan Cancer Hospital and The Affiliated Cancer Hospital of Xiangya School of Medicine, Central South University, Changsha, Hunan, China; 2 Furong Laboratory, Central South University, Changsha, Hunan, China; 3 Computation Biology Department, China National Center for Bioinformation, Beijing, China; 4 Institute of Integrated Traditional Chinese and Western Medicine, Huashan Hospital Fudan University, Shanghai, China

**Keywords:** FLAD1, immunotherapy, necrosis by sodium overload, renal clear cell carcinoma, tumor microenvironment

## Abstract

**Background:**

A new type of regulated cell death known as Necrosis by Sodium Overload (NECSO) has been discovered recently. There is growing evidence indicating that NECSO is essential in both anti-tumor immune responses and the proliferation of cancer cells. Nonetheless, the underlying mechanisms and clinical relevance of NECSO are still not well understood, especially regarding its prognostic significance in kidney renal clear cell carcinoma (KIRC).

**Methods:**

We utilized Non-negative Matrix Factorization (NMF) to distinguish unique NECSO patterns derived from NECSO-associated genes within the TCGA dataset, which led to the identification of three distinct subgroups. Furthermore, we created an innovative NECSO score (NECSOS) utilizing machine learning techniques and confirmed its clinical relevance through validation in several independent datasets, which comprised one transcriptomic cohort of immune checkpoint inhibitor (ICI)-treated KIRC patients, three pan-cancer ICI-treated cohorts, two single-cell RNA sequencing datasets of KIRC patients, and one single-cell dataset from patients treated with PD-1 inhibitors. To characterize FLAD1, we performed gain- and loss-of-function assays for proliferation, migration, and invasion, sodium overload (NC1) sensitivity assays, subcutaneous xenograft models, and CD8+ T cell co-culture cytokine profiling.

**Results:**

The genetic landscape and immune microenvironment of these subgroups were thoroughly characterized, uncovering important insights into the heterogeneity of the tumor microenvironment (TME) and its response to immunotherapy. The NECSO model we developed exhibited strong predictive accuracy for prognosis and immunotherapy responses in patients with KIRC, with validation conducted in diverse pan-cancer ICI cohorts. FLAD1 was identified as a novel prognostic biomarker, and its oncogenic roles in proliferation, migration, and invasion were experimentally confirmed. Mechanistically, FLAD1 regulated cellular sensitivity to TRPM4-mediated sodium overload, promoted tumor growth *in vivo*, and modulated the secretion of T cell-recruiting chemokines and pro-inflammatory cytokines, positioning it as a mechanistic driver within the NECSO-immunity axis rather than merely a prognostic marker.

**Discussion:**

This study has established a robust NECSO-based classification system and prognostic model for KIRC while identifying FLAD1 as a novel biomarker and functional driver of the necrosis-immunity axis. These integrated approaches provide clinically actionable tools for predicting patient outcomes and immunotherapy efficacy.

## Introduction

Renal cell carcinoma (RCC) is the most common type of kidney cancer, with around 210,000 new cases reported each year globally, making up 2-3% of all cancer diagnoses. The primary histological subtype, known as kidney renal clear cell carcinoma (KIRC), constitutes 80-90% of RCC occurrences (1). While surgical resection effectively treats early-stage disease, up to 30% of patients experience recurrence or metastasis following radical surgery, resulting in poor outcomes (2). Metastatic RCC (mRCC) remains incurable, with median survival of only 18 months and dismal 5-year survival rates (3). These clinical challenges underscore the urgent need for novel diagnostic and prognostic biomarkers to guide personalized therapeutic strategies.

Immunotherapy efficacy is governed by complex, interconnected factors within the tumor microenvironment (TME). Key determinants include infiltrating immune cell composition, cytokine profiles, immune checkpoint expression, and MHC-mediated antigen presentation (4–6). However, TME heterogeneity across cancers and patients, driven by underlying genetic diversity, complicates therapeutic predictions (7). While established biomarkers such as tumor mutation burden (TMB) provide valuable insights into cancer-immunity cycle initiation, they exhibit significant limitations (8). Additionally, metabolic reprogramming within the TME impairs immune effector cell function and survival (8). The limitations of existing biomarkers are illustrated by the inability of TMB to forecast the advantages of anti-PD-1 and anti-CTLA-4 combination therapy in various types of cancer (8). These limitations underscore the critical need for robust, integrated biomarker strategies to optimize patient stratification and therapeutic outcomes.

Regulated cell death (RCD) critically influences tumorigenesis, progression, and immunotherapy responses by modulating the tumor microenvironment (9). Over the past decades, several distinct RCD modalities have been characterized, each defined by a specific molecular machinery and biological outcome. Apoptosis, the most extensively studied form, is executed through caspase cascades and typically proceeds in a non-inflammatory, immunologically silent manner. Necroptosis, in contrast, is a lytic form of death driven by the RIPK1-RIPK3-MLKL signaling axis when caspase-8 activity is suppressed, resulting in membrane rupture and the release of damage-associated molecular patterns. Pyroptosis is initiated by inflammasome-activated caspases, which cleave gasdermin D (GSDMD) to form membrane pores, leading to cell swelling and a strongly pro-inflammatory response. Ferroptosis arises from iron-dependent accumulation of lipid peroxides that overwhelm the antioxidant capacity of GPX4, whereas cuproptosis is triggered by intracellular copper overload that induces aggregation of lipoylated tricarboxylic acid cycle proteins and consequent proteotoxic stress.

Necrosis by Sodium Overload (NECSO) represents a recently identified RCD modality that is mechanistically distinct from all of the above pathways (10). Unlike apoptosis, necroptosis, and pyroptosis, NECSO does not depend on caspases, the RIPK1-RIPK3-MLKL axis, or gasdermin-mediated pore formation; and unlike ferroptosis and cuproptosis, it is not driven by iron-catalyzed lipid peroxidation or copper-induced proteotoxicity. Instead, NECSO is uniquely initiated by sustained activation of the TRPM4 cation channel: the selective agonist NC1 binds transmembrane-domain pockets of TRPM4 and locks the channel in an open state, driving uncontrolled Na+ influx, membrane depolarization, osmotic dysregulation, and ultimately necrotic cell death (10, 11). This ion-overload-based mechanism therefore defines NECSO as a unique, TRPM4-dependent form of programmed necrosis that is molecularly and functionally separable from other established cell death pathways. Although recent findings indicate the prognostic significance of NECSO in advanced cancers (12, 13), its effect on tumor microenvironment composition and immunotherapy efficacy in KIRC has not yet been investigated.

In recent years, machine learning has emerged as a powerful approach for deciphering the high-dimensional complexity of cancer transcriptomes. Unlike conventional statistical methods that are often limited to single-variable associations, machine learning can integrate thousands of features simultaneously, uncover hidden molecular subtypes, and distill complex expression patterns into robust and reproducible predictive signatures, thereby enabling more precise patient stratification and treatment guidance. Given these advantages, we adopted an integrated machine learning framework to explore the prognostic and immunological relevance of NECSO in KIRC. Specifically, we first developed a comprehensive NECSO-based classification system to stratify KIRC patients into three molecularly distinct clusters with divergent TME characteristics and immunotherapy responses. Then, to ensure the robustness of feature selection, we performed LASSO regression over 500 iterations, retaining only the genes consistently selected across all runs to minimize the randomness of any single selection. This yielded a robust NECSO prognostic model that reliably predicts patient outcomes and immunotherapy efficacy across KIRC and pan-cancer cohorts. Moreover, functional studies identified FLAD1, a key NECSO model component, as a candidate functional driver and potential therapeutic target. Our integrated approach elucidates the molecular basis of KIRC heterogeneity and provides clinically actionable tools for patient stratification and personalized immunotherapy selection.

## Materials and methods

### Data acquisition and evaluation

Initially, we obtained transcriptome datasets pertaining to KIRC from the TCGA data portal, which comprised 535 tumor samples and encompassed clinical variables including age, gender, disease stage, survival rates, and progression-free survival. Furthermore, somatic mutation information for KIRC was retrieved from the TCGA database. Any samples lacking survival data were omitted from our analysis. The TCGA RNA-seq data were downloaded as raw counts and normalized to transcripts per million (TPM) for downstream analyses.

To explore the KIRC microenvironment, we collected a total of 18 single-cell KIRC tumor samples, comprising 14 tumor samples from the GEO database (accession number: GSE159115) and 4 tumor samples from the GSA-human database (accession number: PRJNA768891). We accessed data regarding chemotherapy responses utilizing the R package TCGAbiolinks (v 2.16.4).

### Immunotherapy-associated dataset collection

Multiple immunotherapy cohorts were assembled to validate NECSO model predictive accuracy. The Braun DA cohort (anti-PD-1-treated advanced ccRCC, n = 181) (14), Riaz N cohort (anti-CTLA-4/PD-1-treated melanoma, GSE91061, n = 101) (15), Wang GY cohort (anti-PD-1/PD-L1-treated melanoma, n = 62) (16), and Jung H cohort (anti-PD-1/PD-L1-treated NSCLC, GSE135222, n = 27) (17) were obtained from published supplementary data or GEO.

Single-cell data from PD-1-treated basal cell carcinoma patients were accessed through GEO (GSE123814, n = 10 tumor samples). Corresponding gene expression profiles and clinical annotations were collected for all cohorts.

### Prediction of immunotherapy response of KIRC

Tumor Immune Dysfunction and Exclusion (TIDE) scores were calculated from normalized KIRC expression data to evaluate PD-1/CTLA-4 immunotherapy efficacy using the TIDE web portal.

### Gene set variation analysis and functional enrichment analysis

The GSVA R package was utilized to carry out Single-sample Gene Set Enrichment Analysis (ssGSEA) in order to measure the enrichment of hallmark and immune-related pathways among NECSO score (NECSOS) patient groups. Gene sets were sourced from MSigDB (http://software.broadinstitute.org/gsea/msigdb/index.jsp). To provide functional annotation for genes associated with NECSO, Gene Ontology (GO) and KEGG pathway enrichment analyses were performed with the clusterProfiler R package. Additionally, tumor purity scores for TCGA-KIRC samples were determined using ESTIMATE (v1.0.13).

### Development of a NECSO model through machine learning analysis

To reduce model complexity, LASSO regression was performed over 500 iterations to eliminate redundancy. Genes appearing in all 500 iterations were selected for model construction using the glmnet R package. Multivariate Cox regression was subsequently applied, with gene combinations stratified and evaluated by area under the curve (AUC). The optimal 13-gene (including C4orf3, CACNB1, CTSO, FLAD1, LDLRAD4, ZMYND19, BHLHE41, DOLPP1, GCDH, HMBS, NDUFV1, NECAB3, TGFA) NECSO model was selected based on maximum AUC performance. The NECSO score for each sample was calculated using the following formula:


$$NECSOS=a_{j}*\sum_{j=1}^{n}\exp_{ji}+\text{β}$$

where exp represents gene expression levels, α denotes the LASSO regression coefficient, and β represents the adjustment coefficient. NECSOS was computed by summing the products of each gene’s expression level and corresponding regression coefficient across all model genes.

### Validation of the model performance

Patients were stratified into high and low NECSOS groups using optimal cutoff values determined by the survminer R package (v0.4.9). Kaplan-Meier survival curves and receiver operating characteristic (ROC) analyses were generated to evaluate prognostic performance. Statistical significance was set at P < 0.05. Chi-squared tests assessed associations between NECSOS groups and immunotherapy response (responder vs. non-responder). Model performance was considered robust when AUC values exceeded 0.7.

### Evaluation of pathway activity using PROGENy

Pathway activity in KIRC patients was assessed using PROGENy R package (18), which infers pathway activities from gene expression data using pathway-responsive genes derived from perturbation experiments.

### Identification of NECSO associated genes

NECSO-associated genes were identified through Pearson correlation analysis with TRPM4, the core mediator of NECSO (10), using gene expression data derived exclusively from tumor samples, with adjacent normal tissue samples excluded to minimize confounding effects of non-malignant tissue. Prior to correlation analysis, gene expression data were preprocessed to ensure analytical quality and biological relevance. Genes with a standard deviation (SD) > 0.1 across tumor samples were retained to exclude lowly variable genes. Additionally, Wilcoxon rank-sum test was applied to identify genes with statistically significant differential expression between tumor and adjacent normal tissue samples (P < 0.05), ensuring that only genes associated with disease progression were included in the subsequent analyses. Pearson correlation coefficients between TRPM4 and all protein-coding genes were calculated, and TRPM4 itself was excluded from downstream analyses to avoid the self-correlation (r = 1, P < 0.001). All protein-coding genes were extracted from the Ensembl database via the biomaRt R package and retained for subsequent analyses. Protein-coding genes with |r| > 0.3 and P < 0.01 were classified as NECSO-associated.

### Cell lines and culture

Two human RCC cell lines, specifically 786-O and 769-P, were sourced from the Cell Bank affiliated with the Chinese Academy of Sciences. For routine maintenance, these cells were grown in RPMI-1640 culture medium (Gibco) enriched with fetal bovine serum at 10% concentration (FBS, Gibco), along with penicillin at 100 units per milliliter and streptomycin at 100 micrograms per milliliter. Standard culture conditions involved keeping all cell lines within a temperature-controlled incubator set to 37 degrees Celsius, maintaining humidity and 5% CO_2_.

### Plasmid construction and establishment of stable cell lines

FLAD1 overexpressing: we amplified the entire coding region of human FLAD1 and subsequently ligated it into a pcDNA3.1(+) expression plasmid (this recombinant construct was termed FLAD1-oe). An empty pcDNA3.1 backbone served as our negative control (designated Vector). For introducing these constructs into 786-O cells, we utilized Lipofectamine 3000 transfection reagent purchased from Invitrogen, strictly adhering to the supplier’s recommended protocol. Transfected cells were allowed to recover for 48 hours before being harvested for downstream functional experiments.

FLAD1 knockdown: For stable knockdown of FLAD1, two specific short hairpin RNA (shRNA) sequences targeting FLAD1 (shFLAD1-1:

Forward 5’

-CCGGCTACAGAGACATCTGGGATTTCTCGAGAAATCCCAGATGTCTCTGTAGTTTTTG-3’. Reverse 5’

-AATTCAAAAACTACAGAGACATCTGGGATTTCTCGAGAAATCCCAGATGTCTCTGTAG-3’ and shFLAD1-2: Forward 5’

-CCGGTCCACTCAAAGGAGCTATATGCTCGAGCATATAGCTCCTTTGAGTGGATTTTTG-3’. Reverse 5’

-AATTCAAAAATCCACTCAAAGGAGCTATATGCTCGAGCATATAGCTCCTTTGAGTGGA-3’) and a non-targeting control shRNA (shCtrl: Forward 5’

-CCGGCAACAAGATGAAGAGCACCAACTCGAGTTGGTGCTCTTCATCTTGTTGTTTTTG-3’. Reverse 5’

-AATTCAAAAACAACAAGATGAAGAGCACCAACTCGAGTTGGTGCTCTTCATCTTGTTG-3’) were cloned into the pLKO.1-puro vector. Lentiviral particles were produced by co-transfecting HEK293T cells with the shRNA constructs together with the packaging plasmids psPAX2 and pMD2.G. 769-P cells were then infected with the harvested lentiviral supernatant. Stable polyclonal cell populations were selected using 1 μg/mL puromycin for 7 days.

### Quantitative real-time PCR

We isolated total cellular RNA utilizing TRIzol extraction reagent purchased from Invitrogen, followed by reverse transcription into complementary DNA through the PrimeScript RT Reagent Kit supplied by TaKaRa. Real-time quantitative PCR amplification was carried out employing SYBR Green-based Master Mix from Roche, with reactions run on an Applied Biosystems StepOnePlus Real-Time PCR instrument. GAPDH served as our housekeeping gene for normalization purposes. The primer sequences were: FLAD1: Forward 5’- TATGGCACAGATCCTTGCACT-3’; Reverse 5’-TGGGAAGAGGTAGACGTTTCG-3’. GAPDH: Forward 5’- CTGGGCTACACTGAGCACC -3’; Reverse 5’- AAGTGGTCGTTGAGGGCAATG -3’Relative mRNA expression levels were calculated using the 2^(-ΔΔCt) method.

### Western blotting

Total cellular proteins were isolated using RIPA lysis buffer (Beyotime), which was augmented with protease inhibitors (Roche). Proteins (30 μg per lane) underwent separation via SDS-PAGE and were subsequently transferred to PVDF membranes (Millipore). The membranes were then blocked with 5% non-fat milk for 1 hour at room temperature, followed by incubation with primary antibodies targeting FLAD1 (1:1000, Proteintech) and GAPDH (1:5000, Proteintech) overnight at 4 °C. After thorough washing, the membranes were treated with HRP-conjugated secondary antibodies (1:5000, Cell Signaling Technology) for 1 hour at room temperature. The signals were detected using an ECL chemiluminescence kit (Thermo Fisher Scientific).

### Cell proliferation assay

Cell proliferation was evaluated utilizing the Cell Counting Kit-8 (CCK-8; Beyotime). Cells that were transfected or infected were placed in 96-well plates at a concentration of 2 × 10³ cells per well. After 1, 2, 3, and 4 days of seeding, 10 μL of CCK-8 solution was added to every well, and the cells were incubated for 2 hours at 37 °C. A microplate reader (Bio-Rad) was used to measure the optical density (OD) at 450 nm. Each experimental group underwent triplicate testing.

### Colony formation assay

Cells were cultured in 6-well plates at a density of 2000 cells per well for a duration of 14 days. Colonies were preserved using 4% paraformaldehyde for 15 minutes, followed by staining with 0.1% crystal violet for 20 minutes, and subsequently rinsed with PBS. Colonies containing 50 or more cells were counted by hand. The experiments were conducted independently on three separate occasions.

### Cell migration and invasion assays

Cellular migration and invasion abilities were assessed utilizing Transwell chambers (8-μm pore size, Corning). In the migration assay, after washing with PBS, 5 × 10^4^ cells were seeded in serum-free medium in the upper chamber. The lower chamber contained medium enriched with 20% FBS, serving as a chemoattractant. Following a 24-hour incubation, any non-migrated cells on the upper surface were carefully removed using a cotton swab. The cells that successfully migrated to the lower membrane surface were subsequently fixed, stained with crystal violet, and photographed. Five random microscopic fields within each insert were counted for analysis.

For the invasion assay, the methodology remained consistent, with the exception that Transwell inserts were pre-coated with Matrigel (1:8 dilution, Corning), and the incubation duration was prolonged to 48 hours.

### Xenograft mouse models

786-O cells in the logarithmic growth phase were harvested by trypsin digestion and counted. After resuspension in sterile saline, an equal volume of Matrigel was added to achieve a final cell concentration of 1 × 107 cells/mL. The cell suspension was maintained on ice until use. Each nude mouse received a subcutaneous injection of 1 × 10^6^; cells in 100 µL of the cell–Matrigel mixture into the dorsal flank. Body weight (g) and tumor volume (calculated as volume = length × short diameter²/2, in mm³) were monitored every three days. When the tumor volume reached approximately 1000 mm³, the mice were euthanized by an intraperitoneal injection of pentobarbital sodium at a dose of 100 mg/kg. After confirming that the mice had died peacefully, the subcutaneous xenograft tumors were excised. Tumor volume and weight were recorded, and the samples were processed for subsequent analyses.

### Cell viability assay

Viable cells were measured using Cell Counting Kit-8 as previously described. Briefly, cells were seeded onto 96-well plates and subsequently treated with NC1. Next, cells were exposed to 10 µL of CCK-8 reagent (100 µL of medium per well) for 2 h at 37 C with 5% CO2 in an incubator. The absorbance was determined at a wavelength of 450 nm.

### ProcartaPlex multiple immunoassay

Cell culture supernatants were collected and centrifuged, then analyzed for multiple cytokines and chemokines using the Human Cytokine and Chemokine 34-Plex ProcartaPlex Panel 1A kit on a Luminex platform. CD8^+^T cells were isolated from healthy donor peripheral blood using the Dynabeads™ Untouched™ Human CD8 T Cells Kit. 786-O and 769-P cells were co-cultured with T cells for 48 h, washed with PBS, stained with crystal violet, and measured at OD 570 nm.

### Statistical analysis

The experimental results are expressed as mean ± SD from a minimum of three independent trials. For statistical analysis, comparisons were conducted using either Student’s t-test for two groups or one-way ANOVA for multiple groups in GraphPad Prism 8.0. Continuous variables among NECSOS groups were compared using Wilcoxon rank-sum tests. The unsupervised clustering of gene expression linked to NECSO was executed with the Non-negative Matrix Factorization (NMF) R package. The analysis and visualization of somatic mutation profiles were carried out using maftools (v1.0.2). Chi-squared tests were employed to evaluate the relationship between clinical features and responses to immunotherapy across the groups. Survival differences among subgroups and NECSOS categories were assessed through Kaplan-Meier survival analysis combined with log-rank tests, and hazard ratios (HRs) were reported with 95% confidence intervals (CIs). For analyses involving multiple comparisons, P values were adjusted using the Benjamini-Hochberg (BH) method to control the false discovery rate (FDR), and an adjusted P value (FDR) < 0.05 was considered statistically significant. A two-sided statistical significance threshold was established at P < 0.05 (*P < 0.05; **P < 0.01; ***P < 0.001) unless otherwise specified.

## Results

### NECSO-associated genotyping of kidney clear cell carcinoma

**Figure 1:**
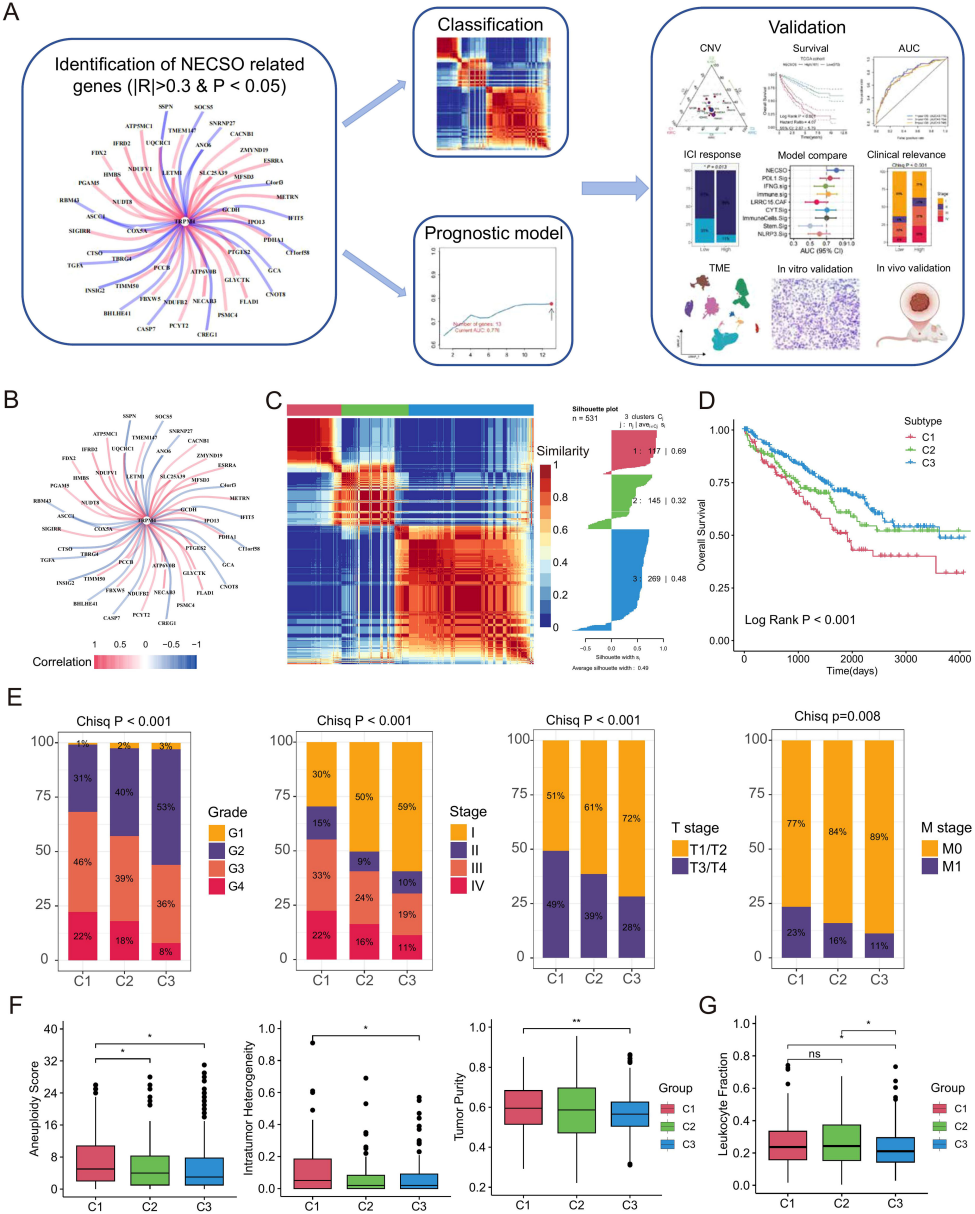
NECSO-associated genotyping of kidney clear cell carcinoma. **(A)** Schematic overview of the study workflow. **(B)** Network diagram showing genes significantly correlated with TRPM4 expression in the TCGA-KIRC cohort, evaluated by Pearson correlation analysis (|R| > 0.3, P < 0.05). Blue edges represent negative correlations and red edges represent positive correlations. **(C)** Consensus connectivity matrix of KIRC patients from the TCGA cohort based on non-negative matrix factorization (NMF) clustering at K = 3. Color intensity reflects the frequency with which two samples were assigned to the same cluster across resampling iterations. **(D)** Kaplan-Meier survival curves of overall survival among the three NMF-defined subtypes in KIRC. Statistical significance was assessed by the log-rank test. **(E)** Stacked bar plots showing the distribution of histological grade, pathological stage, T stage, and M stage across the three subtypes. Differences among groups were evaluated using the chi-square test. **(F)** Box plots comparing aneuploidy score, intratumor heterogeneity, and tumor purity among the three subtypes. In each box plot, the center line indicates the median, box limits represent the interquartile range (IQR), and whiskers extend to 1.5 × IQR. Between-group differences were assessed using the two-sided Wilcoxon rank-sum test. *p < 0.05, **p < 0.01. **(G)** Box plots showing comparisons of leukocyte fraction among the three groups, assessed by the two-sided Wilcoxon rank-sum test. *p < 0.05.

To investigate the prognostic significance of Necrosis by Sodium Overload (NECSO) in KIRC, we identified genes significantly correlated with TRPM4, the core mediator of Necrosis by Sodium Overload (**Figures 1A, B**). GO analysis revealed enrichment in mitochondrial energy metabolism, transmembrane transport, and oxidoreductase pathways, while KEGG analysis highlighted oxidative phosphorylation and metabolic processes (**Supplementary Figures 1A, B**). Non-negative Matrix Factorization (NMF) clustering of NECSO-related gene expression stratified KIRC patients into three distinct subgroups with significant differences in overall survival (OS, P < 0.001), progression-free survival (PFS, P < 0.001), and disease-specific survival (DSS, P < 0.001) (**Figures 1C, D** **Supplementary Figure S1C**). Cluster 3 exhibited the most favorable outcomes, while cluster 1 demonstrated the poorest survival.

Clinical characterization revealed that cluster 1 patients predominantly presented with advanced disease features (G3/G4, Stage III/IV, T3/T4, M1), older age (>65 years), and increased lymph node metastasis, consistent with their poor prognosis (**Figures 1D, E**; **Supplementary Figure S1D**). Conversely, cluster 3 patients exhibited early-stage characteristics aligning with superior survival outcomes. Cluster 1 also demonstrated reduced chemotherapy sensitivity with higher progressive/stable disease rates and elevated malignancy markers including aneuploidy scores, intratumor heterogeneity, and tumor purity (**Figure 1F**; **Supplementary Figure S1E**). Notably, cluster 2 displayed higher leukocyte fractions, suggesting enhanced tumor-suppressive microenvironments potentially favorable for immunotherapy (**Figure 1G**).

These findings establish three molecularly distinct KIRC subgroups: cluster 1 characterized by advanced, treatment-resistant disease; cluster 3 representing early-stage tumors with favorable prognosis; and cluster 2 displaying intermediate features with immunotherapy potential.

### Tumor microenvironment in three clusters

**Figure 2:**
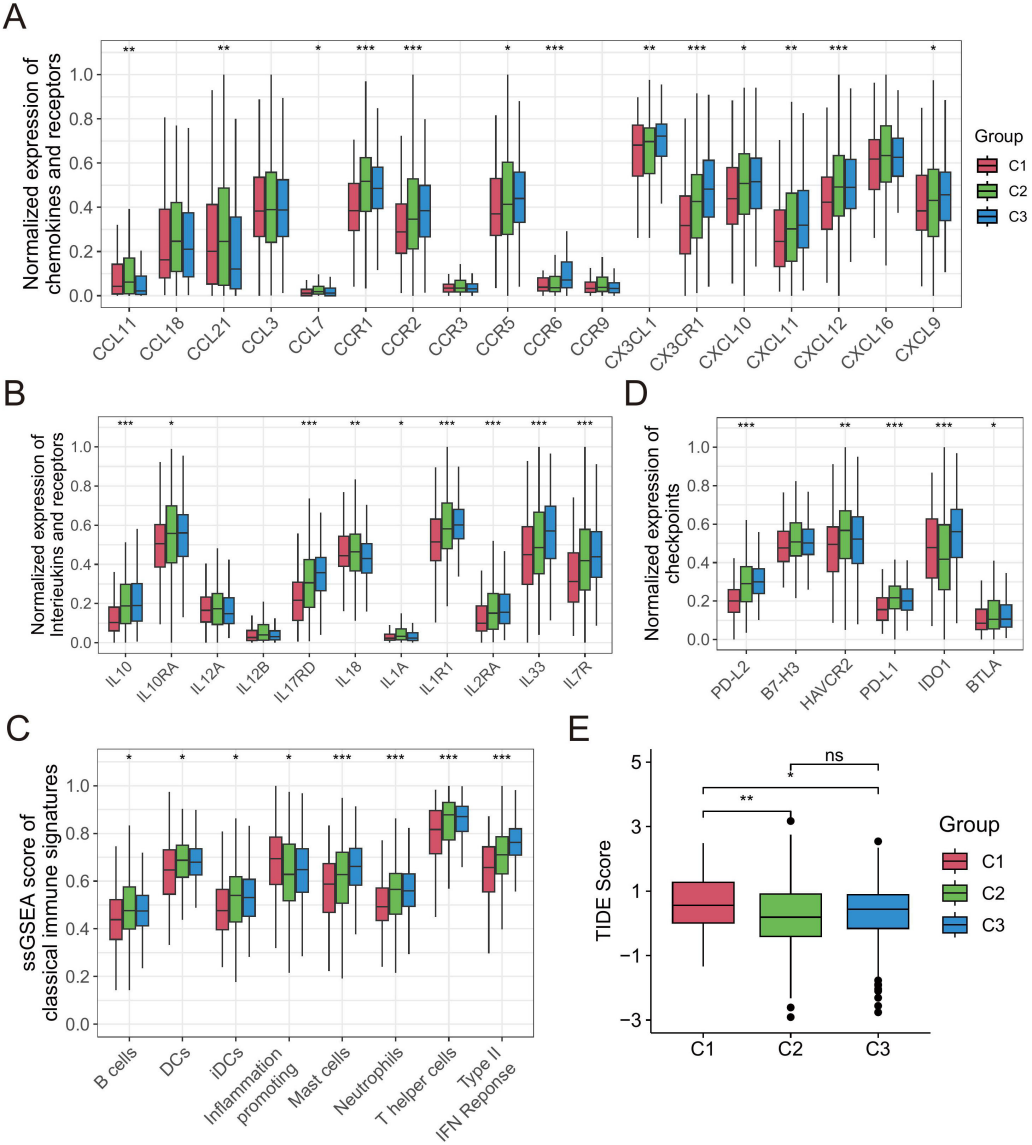
Tumor microenvironment characterization and immunotherapy response prediction across NECSO clusters. **(A)** Box plots comparing normalized expression levels of chemokines and their receptors among the three NECSO clusters in the TCGA-KIRC cohort. In each box plot, the center line indicates the median, box limits represent the IQR, and whiskers extend to 1.5 × IQR. Between-group differences were assessed using the two-sided Wilcoxon rank-sum test. **(B)** Box plots comparing normalized expression levels of interleukins and their receptors among the three clusters, assessed by the two-sided Wilcoxon rank-sum test. **(C)** Box plots comparing single-sample gene set enrichment analysis (ssGSEA) scores of classical immune signatures among the three clusters. ssGSEA scores were calculated using the GSVA R package based on published immune-related gene sets, and between-group differences were assessed using the two-sided Wilcoxon rank-sum test. **(D)** Box plots comparing normalized expression levels of immune checkpoint molecules among the three clusters, assessed by the two-sided Wilcoxon rank-sum test. **(E)** Box plot comparing TIDE scores among the three clusters. TIDE scores were obtained from the TIDE web platform (http://tide.dfci.harvard.edu/), and between-group differences were assessed using the two-sided Wilcoxon rank-sum test. *P < 0.05, **P < 0.01, ***P < 0.001.

Immunotherapy stands out as the foremost therapeutic strategy for advanced cancers, wherein distinct patient subgroups exhibit unique immunoinfiltration scores. Additionally, tertiary lymphoid structures (TLS) serve as significant hubs for immune cells within the TME, prompting us to explore the expression profiles of various chemokines essential for TLS formation. Consequently, we analyzed the tumor microenvironment across three distinct clusters. Our findings demonstrated that the majority of these chemokines were expressed at high levels in clusters 2 and 3. Notably, chemokines such as CCL11, CCL21, CCL7, and CCR1 were primarily prevalent in cluster 2, while CX3CL1, CX3CR1, CXCL10, CXCL11, CXCL12, CXCL16, and CXCL9 exhibited the highest expression levels in cluster 3 (**Figure 2A**). Furthermore, recognizing that numerous interleukins and their associated receptors correlate with immune-stimulating transcripts, our study further examined the levels of these elements across the different clusters. The results indicated that interleukins and their receptors were also more commonly found in clusters 2 and 3 compared to cluster 1, which aligns with the increased levels of chemokines and the augmented infiltration of immune cells within the tumor microenvironment of these clusters (**Figure 2B**). In conclusion, chemokine levels associated with tumor-infiltrating lymphocytes were significantly elevated in clusters 2 and 3 relative to cluster 1, underscoring their role in influencing immune cell infiltration in the tumor microenvironment, consistent with the improved prognosis noted in patients belonging to clusters 2 and 3. Consistently, we noted that several classical immune-associated signatures, including B cells, dendritic cells (DCs), immature dendritic cells (iDCs), mast cells, neutrophils, T helper cells, and Type II IFN response, were primarily observed in clusters 2 and 3 (**Figure 2B**).

Given the essential role of immune checkpoint expression as a cornerstone for therapy utilizing ICIs, we undertook a comprehensive analysis of immune checkpoint expression among the three clusters. Notably, numerous immune checkpoints-such as PD-L2, HAVCR2, PD-L1, IDO1, and BTLA-exhibited elevated expression levels in clusters 2 and 3 as opposed to cluster 1, suggesting that these clusters may demonstrate a greater responsiveness to immunotherapy (**Figure 2D**). In light of these observations, we propose that the different clusters may react variably to distinct immunotherapy approaches. Thus, we advanced our investigation to assess the ICI response in relation to the varied clusters. Our results demonstrated that cluster 1 presented a higher TIDE score in comparison with clusters 2 and 3, implying that patients within cluster 1 are less likely to be responsive to immunotherapy (**Figure 2E**).

In summary, the findings suggest that these three clusters we defined display unique features in the tumor microenvironment. Specifically, the TMEs of clusters 2 and 3 demonstrate a facilitative role for anti-tumor immune responses, along with heightened levels of immune checkpoint protein expression, which enhances their potential for responding to immune checkpoint inhibitors.

### Genomic variation patterns and functional enrichment of different clusters

**Figure 3:**
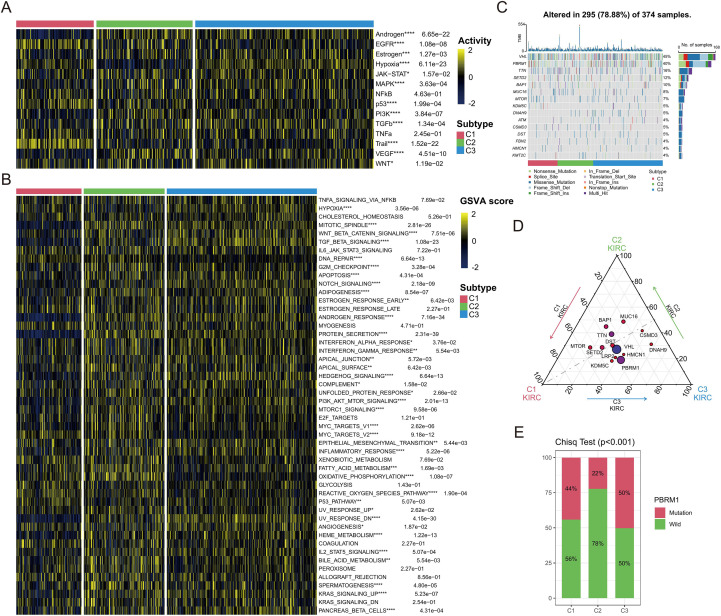
Functional enrichment and genomic variation patterns across NECSO clusters. **(A)** Heatmap showing pathway activity scores across the three NECSO clusters, calculated using the PROGENy algorithm based on perturbation-response gene signatures. Between-group differences for each pathway were assessed using the Kruskal-Wallis test. Color scale represents normalized pathway activity scores. **(B)** Heatmap displaying gene set variation analysis (GSVA) enrichment scores of MSigDB HALLMARK pathways across the three clusters. GSVA scores were computed using the GSVA R package, and between-group differences were assessed using the Kruskal-Wallis test. Color scale represents GSVA enrichment scores. **(C)** Waterfall plot depicting somatic mutation frequencies and mutation types of the top 15 most frequently mutated genes across the three KIRC clusters. Mutation data were obtained from the TCGA-KIRC MAF files and visualized using the maftools R package. The top bar plot indicates the total number of mutations per sample; the right bar plot shows the mutation frequency of each gene. Mutation types are color-coded as indicated in the legend. **(D)** Radar-style plot showing mutation burden of the top 15 mutated genes in each cluster. Circle size represents the mutation frequency of each gene within the corresponding cluster; distance from the cluster center indicates relative mutation load. **(E)** Stacked bar chart showing the proportion of PBRM1-mutated and wild-type samples within each cluster. Differences in mutation distribution among clusters were evaluated using the chi-square test. *P < 0.05, **P < 0.01, ***P < 0.001, ****P < 0.0001.

To elucidate the molecular basis underlying distinct prognostic and immune profiles, we analyzed cancer-related pathway activity using PROGENy. Cluster 1 demonstrated heightened EGFR, p53, hypoxia, and TRAIL pathway activity, consistent with its poor prognosis (**Figure 3A**). GSEA analysis of hallmark pathways revealed that cluster 1 exhibited enrichment in oncogenic signaling (p53 pathway), pro-tumorigenic processes (epithelial-mesenchymal transition), and proliferation pathways (DNA repair, MYC targets v2), providing mechanistic insight into cluster 1’s inferior survival and pro-tumor microenvironment (**Figure 3B**). Conversely, clusters 2 and 3 showed enhanced immune activation signatures, including interferon-α/γ responses and inflammatory pathways.

Somatic mutation analysis of the top 15 most frequently mutated genes revealed VHL and PBRM1 as predominant across all clusters (**Figure 3C**). Cluster-specific patterns emerged: cluster 1 exhibited elevated MTOR mutation frequency, while cluster 2 demonstrated higher MUC16 and BAP1 mutations alongside reduced PBRM1 alterations (**Figures 3D, E**). Notably, PBRM1 deficiency has been shown to reshape the immunosuppressive microenvironment in ccRCC through epigenetic regulation of the PBRM1-KDM5C-IL6 axis (19), consistent with improved survival outcomes in cluster 2 patients with lower PBRM1 mutation rates.

These molecular characterizations establish distinct oncogenic and immune regulatory programs underlying cluster-specific clinical behaviors and therapeutic vulnerabilities.

### Establishment of a NECSO model for predicting the prognosis and immunotherapy response of KIRC patients

**Figure 4:**
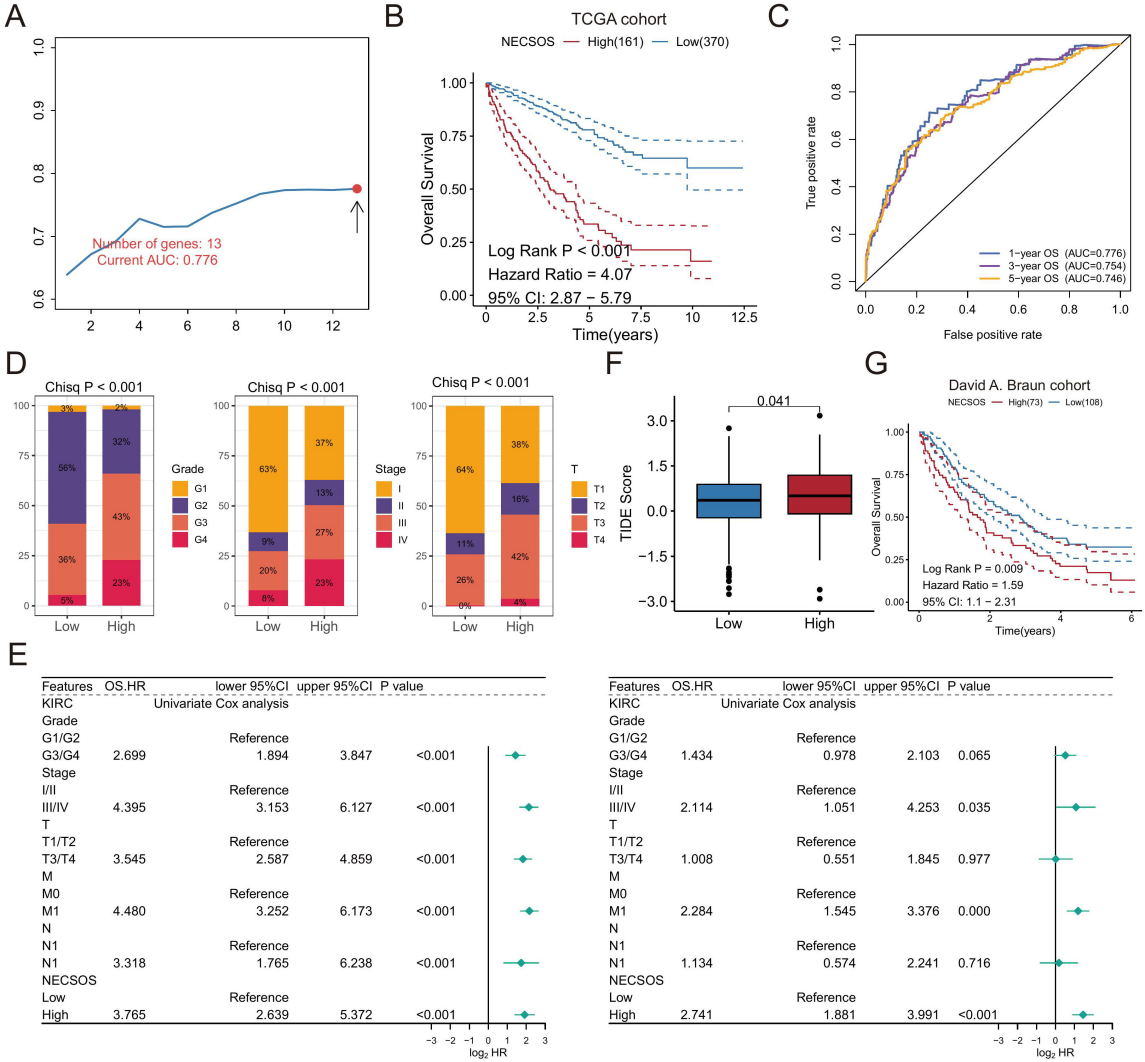
Development and validation of the NECSO prognostic model for KIRC patients. **(A)** Selection of optimal NECSO-related prognostic genes using LASSO-Cox regression. The x-axis represents the number of genes included in the model, and the y-axis represents the area under the curve (AUC). The red dot indicates the optimal model comprising 13 genes with a maximum AUC of 0.776. **(B)** Kaplan-Meier survival curves for TCGA-KIRC patients stratified into high-NECSO (n = 161) and low-NECSO (n = 370) groups based on the NECSOS cutoff value. Between-group survival differences were assessed using the two-sided log-rank test. Hazard ratio (HR) and 95% confidence interval (CI) were derived from univariate Cox regression. **(C)** Time-dependent receiver operating characteristic (ROC) curves evaluating the predictive accuracy of NECSOS for 1-year (AUC = 0.776), 3-year (AUC = 0.754), and 5-year (AUC = 0.746) overall survival in the TCGA-KIRC cohort. **(D)** Stacked bar charts showing the distribution of tumor grade, pathological stage, and T stage between high- and low-NECSOS groups. Differences in clinicopathological feature distribution were evaluated using the chi-square test. **(E)** Forest plots showing univariate (left) and multivariate (right) Cox regression analyses evaluating the independent prognostic value of NECSOS alongside clinicopathological variables (grade, stage, T, M, and N) in KIRC patients. HRs are shown with 95% CIs. **(F)** Box plot comparing TIDE scores between high- and low-NECSOS groups. The center line indicates the median, box limits represent the interquartile range (IQR), and whiskers extend to 1.5 × IQR. Between-group differences were assessed using the two-sided Wilcoxon rank-sum test. **(G)** Kaplan-Meier survival curves for anti-PD-1-treated patients in the David A Braun cohort stratified into high-NECSOS (n = 73) and low-NECSOS (n = 108) groups based on NECSOS. Between-group survival differences were assessed using the two-sided log-rank test. HR and 95% CI were derived from univariate Cox regression.

Having established the association between NECSO and KIRC prognosis and immunotherapy response, we then developed a predictive model using 13 NECSO-related genes through LASSO regression (**Figure 4A**). Patients were stratified into high- and low-NECSO score (NECSOS) groups using optimal cutoffs determined by survminer. Kaplan-Meier analysis demonstrated significantly inferior survival in high-NECSOS patients compared to low-NECSOS patients (**Figure 4B**). ROC analysis confirmed robust predictive performance with AUCs of 0.776 (1-year), 0.754 (3-year), and 0.746 (5-year) survival (**Figure 4C**). Of note, shRNA-mediated knockdown of TRPM4 in 786-O and 769-P cells significantly altered the expression of all 13 signature genes, demonstrating that the model’s predictive power stems from NECSO biology rather than representing a generic proliferation or stress-response signature (**Supplementary Figures 2A-D**; **Supplementary Tables 1, 2**).

NECSOS correlated with disease severity, with higher scores associated with advanced tumor stages (**Figure 4D**). Multivariate Cox regression analysis established NECSOS as an independent prognostic factor superior to conventional clinical parameters including grade, stage, and TNM classification (**Figure 4E**). To evaluate immunotherapy predictive capacity, we compared TIDE scores between NECSOS groups, revealing significantly higher scores in high-NECSOS patients, indicating immunotherapy resistance (**Figure 4F**). Validation in anti-PD-1-treated advanced ccRCC patients confirmed the ability of NECSOS to stratify treatment outcomes, with higher scores predicting worse prognosis (**Figure 4G**).

These findings establish NECSOS as a clinically actionable biomarker for predicting both survival outcomes and immunotherapy efficacy in KIRC patients, demonstrating superior performance compared to standard clinical features.

### NECSO score as a promising predictor for immunotherapy outcomes in pan-cancer cohorts

**Figure 5:**
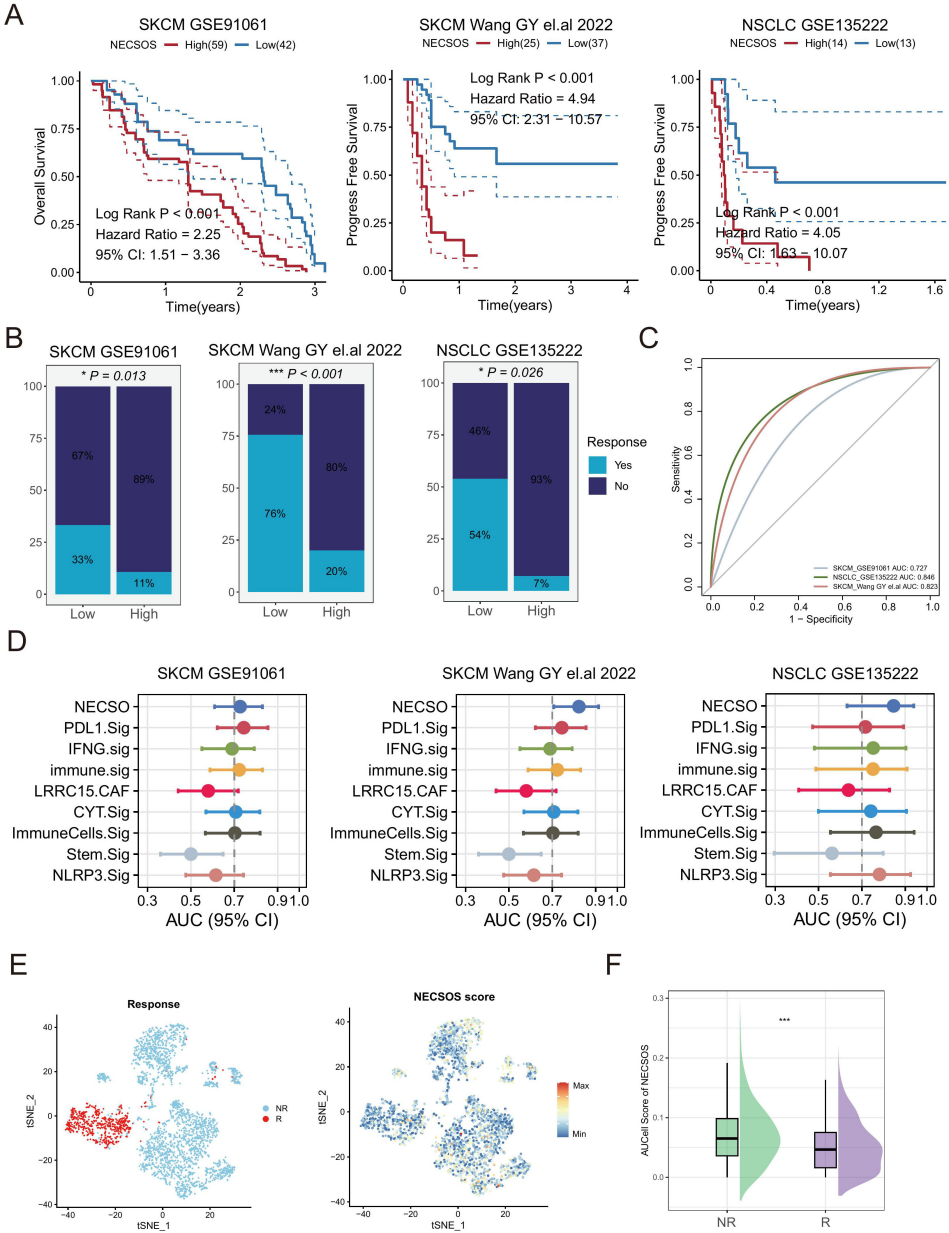
Pan-cancer validation of NECSOS as a predictive biomarker for immunotherapy response. **(A)** Kaplan-Meier survival curves showing overall survival (OS) or progression-free survival (PFS) of immunotherapy-treated patients stratified by high and low NECSOS in melanoma (SKCM; GSE91061, n = 101; Wang GY et al., 2022, n = 62) and non-small cell lung cancer (NSCLC; GSE135222, n = 27). Between-group survival differences were assessed using the two-sided log-rank test. Hazard ratios (HRs) and 95% confidence intervals (CIs) were derived from univariate Cox regression. Dashed lines indicate 95% CIs for each group. **(B)** Stacked bar charts showing immunotherapy response rates stratified by NECSOS groups across the three cohorts. Response was defined as complete response (CR) or partial response (PR); non-response was defined as progressive disease (PD) or stable disease (SD). Differences in response distribution between groups were evaluated using the chi-square test. **(C)** Receiver operating characteristic (ROC) curves evaluating the predictive accuracy of NECSOS for treatment response across multiple ICI cohorts. **(D)** Forest plots comparing the AUC performance of NECSOS with established immunotherapy predictive signatures (PDL1.Sig, IFNG.Sig, immune.Sig, LRRC15.CAF, CYT, ImmuneCells.Sig, Stem.Sig, and NLRP3.Sig) across the three ICI cohorts. Dots represent AUC values; horizontal lines indicate 95% CIs. **(E)** t-distributed stochastic neighbor embedding (t-SNE) plot of malignant cells categorized by treatment response phenotype (left). t-SNE plot showing AUCell scores of NECSO-related gene signatures in malignant cells, with red indicating higher scores and blue indicating lower scores (right). **(F)** Boxplot comparing AUCell scores of NECSO-related gene signatures between non-responder and responder patients in the anti-PD-1 treatment cohort. Box boundaries represent 25th and 75th percentiles, with median values indicated by center lines. *P < 0.05, ***P < 0.001.

To validate NECSOS’s predictive performance across cancer types, we analyzed multiple PD-1/PD-L1 immunotherapy datasets. Consistently, patients with higher NECSOS demonstrated significantly reduced overall survival and progression-free survival following immunotherapy in melanoma (SKCM) and non-small cell lung cancer (NSCLC) cohorts (**Figure 5A**). Treatment response analysis revealed that high-NECSOS patients predominantly exhibited progressive or stable disease, while low-NECSOS patients achieved complete or partial responses (**Figure 5B**). NECSOS demonstrated robust predictive performance with AUC values exceeding 0.7 across all examined cohorts (**Figure 5C**).

Comparative analysis against established immunotherapy response signatures revealed NECSOS’s superior performance over IFNG.Sig (20), Immune.Sig (20), ImmuneCells.Sig (15), PDL1.Sig (21), LRRC15.CAF.Sig (22), NLRP3.Sig (14), Stem.Sig (23), and CYT.Sig (24) across multiple cohorts, while most existing signatures showed optimal performance in only one or two datasets (**Figure 5D**). Single-cell validation using PD-1-treated basal cell carcinoma (BCC) patients confirmed that non-responders exhibited higher frequencies of cancer cells with elevated NECSOS, with significantly higher overall NECSOS compared to responders (P < 0.001, **Figures 5E, F**).

These findings establish NECSOS as a pan-cancer predictor of immunotherapy efficacy, with elevated scores associated with treatment resistance and poor outcomes, while lower scores correlate with enhanced therapeutic benefit and improved survival.

### FLAD1 servers as a novel biomarker and promotes the proliferation and invasion abilities of KIRC

**Figure 6:**
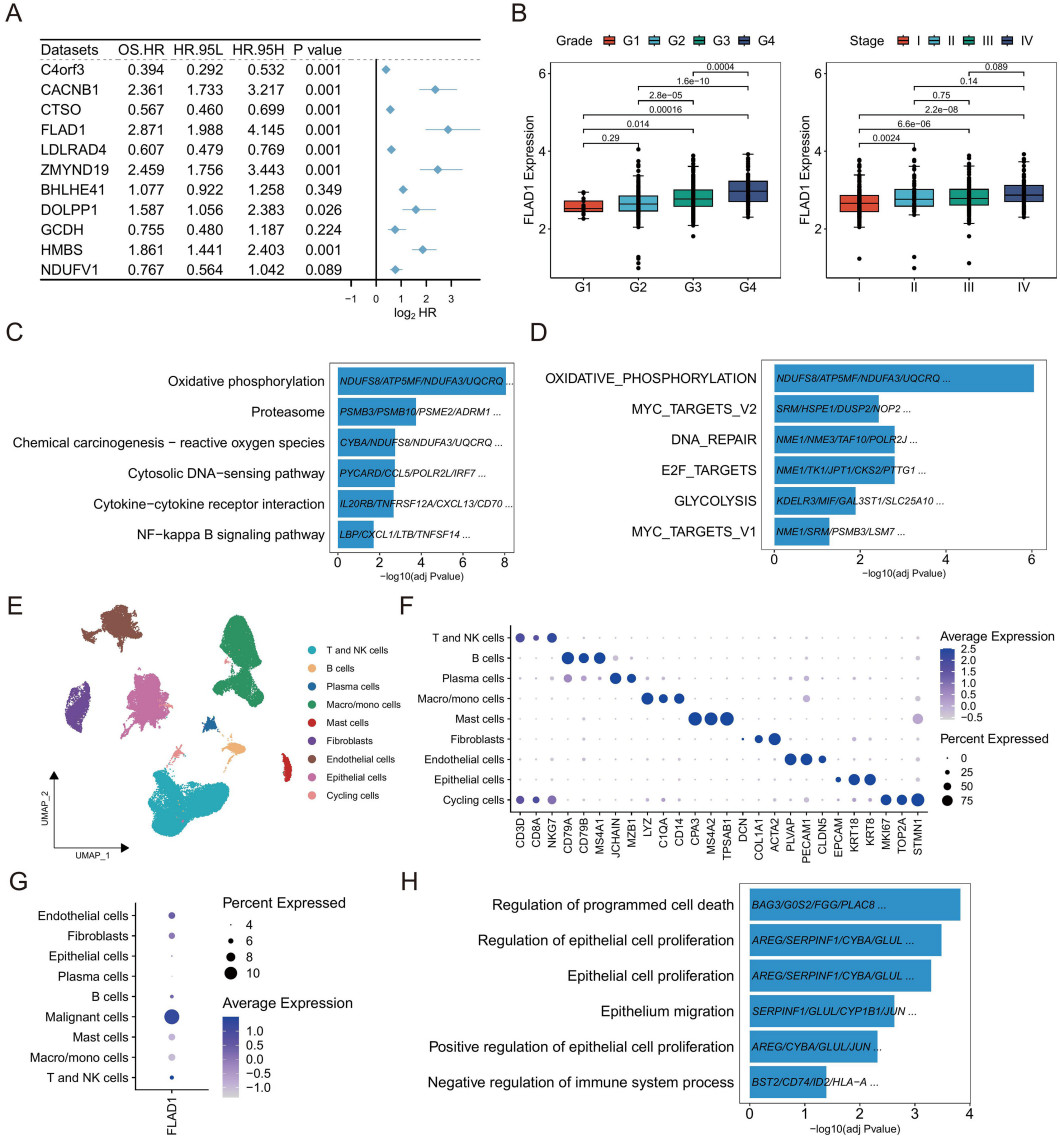
FLAD1 expression correlates with KIRC prognosis and promotes malignant phenotypes. **(A)** Forest plot from univariate Cox regression analysis evaluating the prognostic value of NECSO model genes in KIRC patients. **(B)** Boxplots comparing FLAD1 expression across different tumor grades and pathological stages. **(C, D)** Functional enrichment analysis of high versus low FLAD1-expressing patients using KEGG **(C)** and HALLMARK **(D)** gene sets. **(E)** Uniform Manifold Approximation and Projection (UMAP) plot showing the composition of nine major cell types in KIRC single-cell cohorts. **(F)** Dot plot displaying expression of cell type-specific markers across identified cell populations. **(G)** Dot plot showing FLAD1 expression across cell types, with epithelial cells subdivided into malignant and normal populations based on inferCNV analysis. **(H)** Bar plot showing Gene Ontology (GO) enrichment pathways differentially activated between FLAD1^+^ and FLAD1^−^ malignant cells.

To validate the clinical utility of individual NECSO model components, we performed univariate Cox regression analysis of constituent genes. C4orf3, CTSO, and LDLRAD4 were associated with favorable prognosis, while CACNB1, FLAD1, ZMYND19, DOLPP1, and HMBS correlated with poor outcomes in KIRC patients (**Figure 6A**). Notably, among poor-prognosis genes, only FLAD1 specifically correlated with tumor grade, stage, and T-stage advancement (**Figure 6B**; **Supplementary Figure S3A-C**). Previous studies demonstrated that FLAD1 copy number amplification promotes triple-negative breast cancer progression through lipid metabolism (25) and enhances hepatocellular carcinoma progression by conferring ferroptosis resistance and inhibiting CD8+ T cell infiltration (26).

KEGG enrichment analysis revealed that high FLAD1 expression activated oxidative phosphorylation, proteasome, cytosolic DNA-sensing, chemical carcinogenesis-ROS, NF-κB signaling, and cytokine-receptor interaction pathways. HALLMARK analysis confirmed activation of proliferation and metastasis pathways, including MYC targets, DNA repair, E2F targets, and glycolysis (**Figures 6C, D**).

Single-cell RNA sequencing of 18 KIRC tissues identified nine distinct cell populations: T/NK cells (CD3D, CD8A, NKG7), B cells (CD79A, CD79B, MS4A1), plasma cells (JCHAIN, MZB1), macrophages/monocytes (LYZ, C1QA, CD14), mast cells (CPA3, MS4A2, TPSAB1), fibroblasts (DCN, COL1A1, ACTA2), endothelial cells (PLVAP, PECAM1, CLDN5), epithelial cells (EPCAM, KRT18, KRT8), and cycling cells (MKI67, TOP2A, STMN1) (**Figures 6E, F**). InferCNV analysis distinguished malignant from normal epithelial cells (**Supplementary Figure 3D**), revealing significantly elevated FLAD1 expression in malignant cells (**Figure 6G**). FLAD1+ malignant cells specifically activated epithelial growth and migration pathways while affecting cell death processes, confirming FLAD1’s role in promoting proliferation, invasion, and metastasis (**Figure 6H**). Multivariate Cox regression established FLAD1 as an independent prognostic factor superior to conventional clinical parameters (**Supplementary Figures 3E, F**), with pan-cancer analysis confirming its negative prognostic impact across multiple tumor types (**Supplementary Figure 3G**).

### FLAD1 promotes malignant phenotypes of renal cancer cells

**Figure 7:**
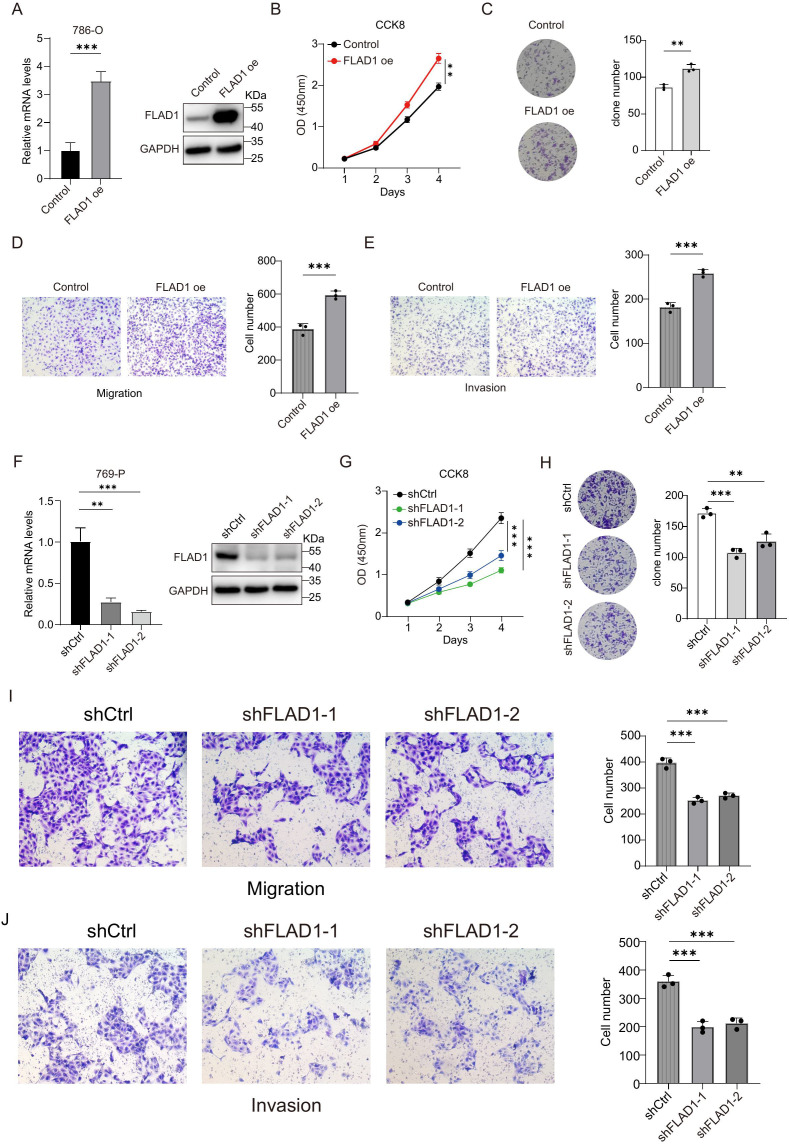
FLAD1 promotes malignant phenotypes in renal cell carcinoma cells. **(A)** qRT-PCR and Western blot analysis of FLAD1 expression in 786-O cells after transfection with FLAD1-overexpressing plasmid (FLAD1-oe) or empty vector control (Vector). GAPDH served as the loading control. **(B)** Cell proliferation of 786-O cells measured by CCK-8 assay over 4 days. **(C)** Colony formation assay of 786-O cells after 14 days of culture. Representative images (left) and quantification (right) are shown. **(D)** Migration ability of 786-O cells evaluated by transwell assay after 24 hours. **(E)** Invasion ability of 786-O cells assessed by matrigel-coated transwell assay after 48 hours. **(F)** qRT-PCR and Western blot analysis of FLAD1 expression in 769-P cells stably expressing shFLAD1-1, shFLAD1-2, or non-targeting shRNA control (shCtrl). GAPDH served as loading control. **(G)** CCK-8 proliferation assay of 769-P cells over 4 days. **(H)** Colony formation assay of 769-P cells. Representative images and quantitative results are presented. **(I)** Migration ability of 769-P cells evaluated by transwell assay after 24 hours. **(J)** Invasion ability of 769-P cells assessed by matrigel-coated transwell assay after 48 hours. *P < 0.05, **P < 0.01, ***P < 0.001.

To investigate the functional role of FLAD1 in renal carcinogenesis, we performed complementary gain- and loss-of-function studies in two RCC cell lines. In 786-O cells with low endogenous FLAD1 expression, stable overexpression was confirmed by qRT-PCR and Western blot (**Figure 7A**). FLAD1 overexpression significantly enhanced malignant phenotypes, including accelerated proliferation (**Figure 7B**), increased colony formation (**Figure 7C**), and enhanced migration (**Figure 7D**) and invasion (**Figure 7E**) compared to controls.

Conversely, FLAD1 knockdown in 769-P cells, which express higher basal FLAD1 levels, was achieved using two independent shRNAs with efficient suppression confirmed by qRT-PCR and Western blot (**Figure 7F**). FLAD1 depletion markedly suppressed proliferation (**Figure 7G**), colony formation (**Figure 7H**), migration (**Figure 7I**), and invasion (**Figure 7J**), with consistent effects observed across both shRNA constructs.

These complementary functional studies establish FLAD1 as a critical driver of proliferation and metastatic potential in renal cancer cells, confirming its oncogenic role and potential as a prognostic biomarker in KIRC.

### FLAD1 modulates sensitivity to sodium overload, tumor growth, and immune-related cytokine profiles

**Figure 8:**
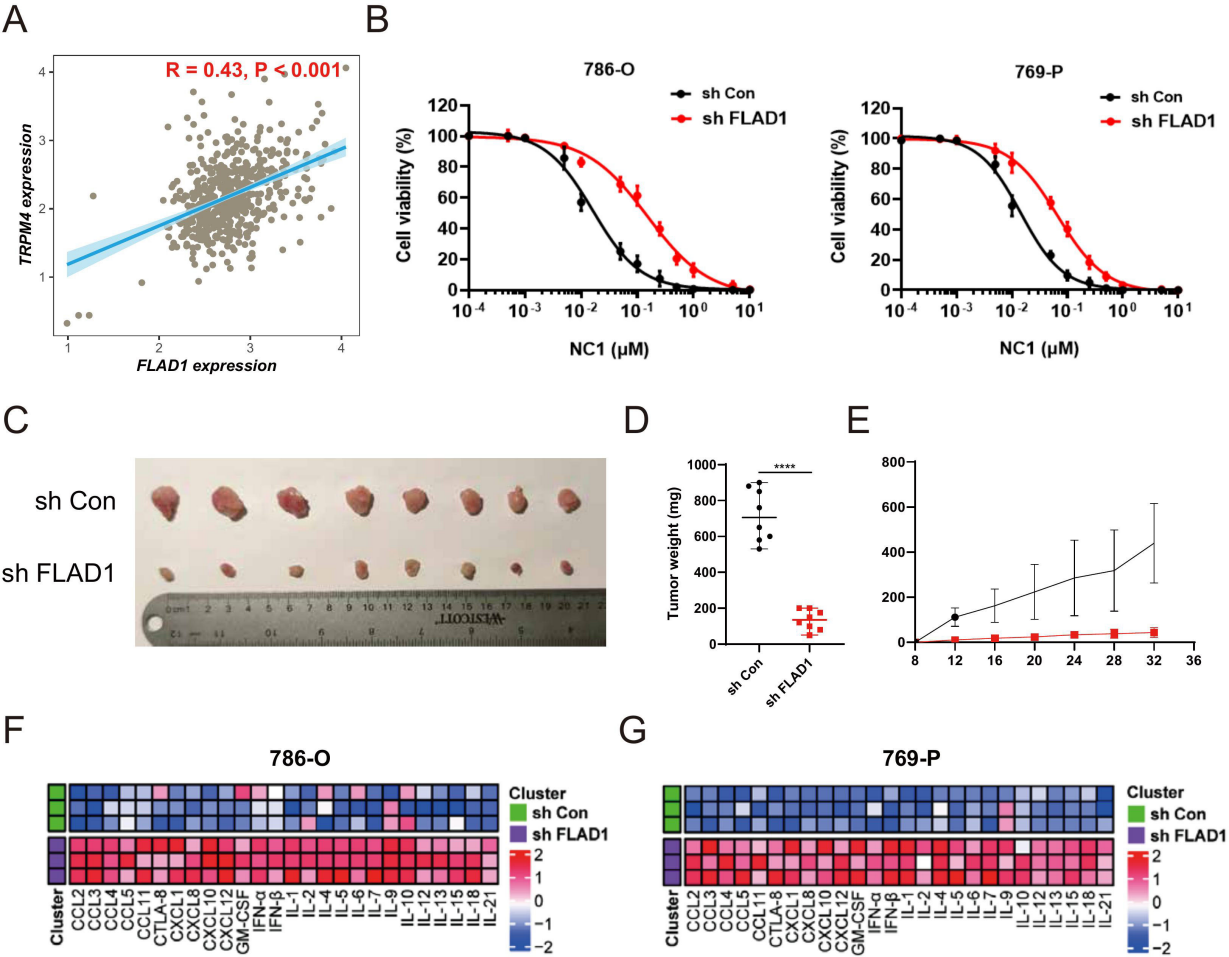
FLAD promotes cells proliferation *in vivo* and knockdown sensitizes renal cell carcinoma cells to Necrosis and immunotherapy. **(A)** Pearson correlation between the expression of TRPM4 and FLAD1 in TCGA-KIRC dataset. **(B)** Dose response curves of NC1 in 786-O and 769-P cells. **(C)** Representative images of xenograft tumors from the shFLAD1 group and the control group in the nude mouse tumor model. **(D)** Tumor weight of xenograft tumors from respective groups. ****P<;0.0001 **(E)** Tumor growth curves from respective groups. **(F, G)** Heatmap of various cytokines and chemokines detected by ProcartaPlex multiple immunoassays between FLAD1 knockdown and the negative control groups in the culture supernatants from 786-O **(F)** and 769-P **(G)** cells.

To address whether FLAD1 functions as a mechanistic driver within the necrosis-immunity axis rather than merely a prognostic marker, we performed a series of functional experiments. First, bioinformatic analysis of TCGA-KIRC data revealed a significant positive correlation between FLAD1 and TRPM4 expression (R = 0.43, P < 0.001), a key channel mediating sodium overload-induced necrotic cell death, suggesting a potential functional link between FLAD1 and the necrotic signaling pathway (**Figure 8A**). To functionally validate this association, we treated FLAD1-knockdown (shFLAD1) and control (shCon) 786-O and 769-P cells with NC1, a selective TRPM4 activator that triggers sodium overload. FLAD1 knockdown resulted in a significant rightward shift of the dose-response curve and a markedly elevated IC50 value in both cell lines compared to controls, indicating that FLAD1 knockdown confers resistance to NC1-induced necrotic stimuli (**Figure 8B**). These data demonstrate that FLAD1 expression positively regulates cellular sensitivity to sodium overload-mediated necrotic cell death.

To further evaluate the *in vivo* relevance of FLAD1, we performed subcutaneous xenograft experiments. Tumors derived from shFLAD1 cells were significantly smaller in size and weight compared to shCon tumors (**Figures 8C, D**), and tumor growth curves confirmed sustained suppression of tumor progression following FLAD1 knockdown (**Figure 8E**). These results extend the pro-tumorigenic role of FLAD1 beyond *in vitro* proliferation and migration phenotypes.

Given the superior predictive performance of the NECSO model in predicting immunotherapy outcomes, we next investigated whether FLAD1 modulates the cytokine secretion profile, which is critical for shaping the tumor immune microenvironment. To this end, FLAD1-knockdown (shFLAD1) and control (shCon) 786-O and 769-P cells were co-cultured with activated human CD8^+^ T cells for 48 hours, after which the conditioned media were collected for multiplex cytokine and chemokine profiling using ProcartaPlex immunoassays. The analysis revealed coordinated induction in cytokine and chemokine expression upon FLAD1 knockdown in both 786-O and 769-P cells (**Figures 8F, G**). Notably, the knockdown of FLAD1 markedly increased the secretion of chemokines involved in the recruitment of T cells and effector immune cells (CCL2, CCL3, CCL4, CCL5, CXCL10, and CXCL12), pro-inflammatory and effector cytokines (IL-1, IL-2, IL-6, IL-12, IL-15, IL-18), as well as type I interferons (IFN-α and IFN-β) and GM-CSF. This secretory phenotype is characteristic of an “immune-hot” microenvironment favorable for cytotoxic T cell recruitment and activation. Collectively, these findings support FLAD1 not merely as a prognostic marker, but as a mechanistic driver linking necrotic cell death sensitivity and tumor immune regulation within the NECSO-immunity axis.

## Discussion

Checkpoint blockade therapy has become established as standard second-line management for advanced malignancies, with expanding adoption in first-line settings. Necrosis by Sodium Overload, characterized by abnormal sodium accumulation leading to programmed cell death, represents a form of cell necrosis triggered by NC1 through its action on TRPM4 to promote sodium ion influx (10). Emerging evidence links NECSO to tumor microenvironment remodeling and antitumor immunity across multiple advanced malignancies (12, 13). Nevertheless, direct evidence establishing the relationship between NECSO and immune checkpoint inhibitor response in KIRC remains lacking.

Consequently, we performed an extensive multi-omic analysis to explore the functional variations within NECSO subclusters and their influence on the immune landscape, alongside possible immunotherapeutic advantages. This study considerably deepens our comprehension of NECSO in the realm of onco-immunology by pinpointing populations with the greatest efficacy potential and formulating effective treatment approaches. Patients with KIRC were sorted into three groups according to NECSO-associated genes, uncovering notable differences in survival, with cluster 3 presenting the best outcomes and cluster 1 demonstrating the least favorable results.

Immune cells within the tumor microenvironment are essential for tumor development, with varying immune cell subtypes associated with different types of tumors. Additionally, immune cell subpopulations can differ among patients even within the same pathological categories (27). In our study, clusters 2 and 3 displayed a clearly inflamed, “immune-hot” phenotype, characterized by enriched infiltration of B cells, dendritic cells, and T helper cells, together with elevated expression of chemokines, interleukins, and immune checkpoint molecules. This profile is particularly relevant given that, although immunotherapy research has historically focused on T cells, mounting evidence indicates that tumor-infiltrating B cells and plasma cells play crucial synergistic roles in tumor control (28), and reduced inflammatory features have been associated with resistance to anti-PD-1 therapy (29). The concurrent upregulation of immune checkpoints in clusters 2 and 3, together with their significantly lower TIDE scores—an established indicator of immune evasion and ICI efficacy (30)—collectively suggest that these patients are more likely to benefit from checkpoint blockade therapy (31). In contrast, cluster 1 exhibited an “immune-cold” microenvironment with suppressed immune function and enhanced pro-tumorigenic activity, which likely explains its poorer prognosis, possibly driven by activation of p53, hypoxia, EMT, and proliferation signaling pathways (32–34). Beyond the immune landscape, distinct somatic mutation patterns further reinforced these subtype-specific differences; notably, cluster 2 harbored a lower frequency of PBRM1 mutations, a gene whose truncating mutations have previously been linked to prolonged survival following PD-1 blockade in the CM-009 cohort (35), providing additional genomic support for the differential immunotherapy responsiveness across NECSO clusters.

Furthermore, we constructed a NECSO model aimed at predicting the prognosis of KIRC and the response to immunotherapy, showing impressive AUC results among KIRC patients. The Kaplan-Meier analysis indicated that individuals with higher NECSOS experienced significantly lower survival rates and more advanced tumor grades when contrasted with those who had lower NECSOS. Additionally, the NECSOS model can serve as a standalone prognostic indicator, effectively forecasting the response to immunotherapy in KIRC, which was validated across various pan-cancer cohorts. Notably, the NECSO model demonstrated superior performance in predicting ICI response relative to several established models, achieving a mean AUC greater than 0.7. Finally, we confirmed that FLAD1, rarely reported in previous KIRC studies, associated with worse patient survival. Moreover, FLAD1 overexpression significantly promoted proliferation and invasion abilities of renal cancer cells, suggesting its potential as a therapeutic target for KIRC.

To provide phenotypic evidence linking FLAD1 to tumor immune regulation, we performed a co-culture experiment in which FLAD1-knockdown and control renal cancer cells were co-cultured with activated CD8+ T cells. FLAD1 silencing significantly enhanced the secretion of chemokines involved in the recruitment of T cells and effector immune cells, supporting a functional association between FLAD1 and antitumor immune responses. As FLAD1 encodes flavin adenine dinucleotide synthetase, a key regulator of cellular metabolism and redox homeostasis, we speculate that it may influence antitumor immunity through metabolic–immune crosstalk. However, we acknowledge that the precise molecular mechanisms by which FLAD1 modulates the tumor immune microenvironment remain to be elucidated, and dedicated mechanistic studies will be required to clarify this issue in future work.

In summary, our study systematically characterized the transcriptomic landscape of NECSO in KIRC, integrating multi-omic analyses of genetic alterations and tumor immune cell infiltration. Based on these findings, we developed an innovative NECSO-based classification system and a robust prognostic model, providing practical tools for individualized prognosis assessment and immunotherapy response prediction in KIRC patients. Additionally, FLAD1 was identified as a novel prognostic biomarker, with its overexpression demonstrating oncogenic functions *in vitro*.

Nevertheless, several limitations of this study should be acknowledged. Most importantly, we acknowledge that the association between FLAD1 and immunotherapy response currently relies on bioinformatic inference, and the underlying mechanisms by which FLAD1 may modulate the tumor immune microenvironment remain to be experimentally validated. Our functional validation was restricted to *in vitro* co-culture systems, and *in vivo* confirmation of immune cell infiltration was not performed; such characterization will require immunocompetent syngeneic or humanized mouse models, as conventional xenografts in immunodeficient hosts lack an intact adaptive immune system, and represents an important direction for future work. Meanwhile, the precise molecular relationship between FLAD1 and TRPM4 remains undefined. In addition, our findings are derived from retrospective analyses of publicly available cohorts and have not yet been validated in a prospective clinical setting. As retrospective designs are inherently subject to selection bias and potential confounding, and the analyzed cohorts may not fully represent the diversity of the broader patient population, the prognostic and predictive value of NECSOS should be interpreted with caution. Moreover, because the stratification thresholds were established using the same datasets, their generalizability requires confirmation in independent populations. Hence, dedicated prospective, multi-center studies recruiting KIRC patients receiving immunotherapy are needed to confirm the clinical utility of NECSOS in prospectively collected, independent cohorts.

## Data Availability

The original contributions presented in the study are included in the article/**Supplementary Material**. Further inquiries can be directed to the corresponding authors.

## Supplementary material

Supplementary Figure 1 Functional annotation of NECSO-related genes and clinical characterization of NECSO clusters. **(A)** GO enrichment analysis of NECSO-related genes. **(B)** KEGG pathway enrichment analysis of NECSO-related genes. **(C)** Kaplan-Meier survival curves for progression-free survival (left) and disease-specific survival (right) across NECSO clusters in KIRC patients. **(D)** Distribution of N stage and patient age across the three clusters. **(E)** Distribution of chemotherapy response rates across the three clusters.

Supplementary Figure 2 TRPM4 knockdown modulates the expression of NECSO signature genes in renal cancer cells. **(A, B)** Relative mRNA expression of TRPM4 and 13 NECSO signature genes in 786-O cells following shRNA-mediated knockdown of TRPM4 (sh TRPM4) compared to control (shCon). Genes negatively correlated with TRPM4 (C4orf3, CTSO, LDLRAD1, and TGFA) are shown in **(A)**, and genes positively correlated with TRPM4 (CACNB1, FLAD1, ZMYND19, BHLHE41, DOLPP1, GCDH, HMBS, NDUFY1, and NECAB3) are shown in **(B)**. **(C, D)** Relative mRNA expression of the same gene sets in 769-P cells upon TRPM4 knockdown, presented as in **(A, B)**, respectively. Data are presented as mean ± SD (n = 3). Statistical significance was determined by unpaired two-tailed Student’s t-test. *p < 0.05, **p < 0.01, ***p < 0.001, ****p < 0.0001.

Supplementary Figure 3 Clinical significance of NECSO-related genes and FLAD1 validation in KIRC. **(A-C)** Boxplots comparing expression levels of poor-prognosis NECSO-related genes across different tumor grades **(A)**, pathological stages **(B)**, and T stages **(C)**. **(D)** Heatmap displaying copy number variations in reference cells and epithelial cells from inferCNV analysis. **(E, F)** Forest plots from univariate **(E)** and multivariate **(F)** Cox regression analyses evaluating the independent prognostic value of FLAD1 in KIRC patients. **(G)** Forest plot from univariate Cox regression analysis evaluating the prognostic value of FLAD1 across pan-cancer cohorts.
